# Integration of transcriptomic and metabolomic analysis of the mechanism of dietary *N*-carbamoylglutamate in promoting follicle development in yaks

**DOI:** 10.3389/fvets.2022.946893

**Published:** 2022-08-29

**Authors:** Jia Zhou, Shuangming Yue, Jingjing Du, Benchu Xue, Lizhi Wang, Quanhui Peng, Huawei Zou, Rui Hu, Yahui Jiang, Zhisheng Wang, Bai Xue

**Affiliations:** ^1^Institute of Animal Nutrition, Sichuan Agricultural University, Chengdu, China; ^2^Department of Bioengineering, Sichuan Water Conservancy College, Chengdu, China; ^3^College of Animal Science and Technology, Sichuan Agricultural University, Chengdu, China

**Keywords:** metabolomic, *N*-carbamoylglutamate, ovarian follicular development, yak (*Bos grunniens*), transcriptomic (RNA-seq)

## Abstract

Yak is the main livestock in the highlands of China. The low reproductive rate of yaks is a serious constraint on their production and utility. *N*-carbamylglutamate (NCG) can increase arginine synthesis in mammals and has been shown to improve reproductive performance. Twelve multiparous and simutaneous anoestrous female yaks were randomly divided into two groups, one of which was fed the basal diet (Control, *n* = 6), and the other was fed the basal diet supplemented with NCG at 6 g/day/yak (NCG, *n* = 6). All yaks were slaughtered on the 32nd day (the time predicted for the selection of the last wave of dominant follicles), and their ovarian tissues were collected and follicles were classified. NCG supplementation increased the number of large ovarian follicles (diameter > 10 mm), as well as caused significant changes in the transcriptional and metabolic levels in yak ovaries which due to the differential expression of 889 genes and 94 metabolites. Integrated analysis of the transcriptomics and metabolomics data revealed that the differentially expressed genes and differential metabolites were primarily involved in the process of energy metabolism, amino acid metabolic pathways, carbohydrate metabolic pathways, and lipid metabolic pathways. The highlighted changes were associated with amino acid synthesis and metabolism, ovarian steroid hormone synthesis, the pentose phosphate pathway, and the tricarboxylic acid cycle, suggesting that NCG supplementation may promote estrogen synthesis and help regulate follicular development by altering the pathways associated with glucose catabolism. The results present important clues for understanding the mechanisms by which NCG supplementation promotes follicular development in yaks. The findings of this study provide a basis for the development and application of NCG in optimizing animal reproduction, including yak reproductive performance, which may help optimize livestock management and uplift the pastoral economy.

## Introduction

Yak is the traditional livestock in the highlands of China. It provides local herders with the most basic means of living and livelihood materials such as meat, milk, shelter, and fuel (dung) (1, 2). However, yaks exhibit low reproductive efficiency, which is attributable to their seasonal reproductive characteristics (breeding period: July–November; when lush pastures provide sufficient nutrition for yaks) has severely constrained the development of yak farming (3). In addition, a large proportion of yaks go through a long postpartum anestrous period, as the next estrous cycle starts the following year or 2 years later rather than the breeding season of the year of calving (4). Hence, most yaks calve twice every 3 years or once every 2 years. The average annual reproductive rate for female yaks at reproductive age has been reported to be <60% (3). In domestic beef cows, uterine repair is achieved within 1 month of successful calving, following which, the cows become ready for the next estrous cycle (5). Approximately 90% of the cows complete their first postpartum ovulation within 6 weeks of calving (6). The duration of the postpartum anestrous period is closely related to the time of ovarian cyclic recovery, that is, the time required for the establishment of dominant follicles and recruitment of new follicles (7). Therefore, accelerating the recovery of postpartum follicular activity can potentially be an effective strategy to shorten the postpartum resting period and improve the fertility of yaks.

The mammalian ovary contains follicles at various stages of development. The primary function of the follicle is to differentiate and release mature oocytes for fertilization and successful continuation of species (8). The process of ovarian follicular maturation involves the processes of enlargement of the oocyte, proliferation of follicular granulosa cells presented around the oocyte, differentiation of the surrounding mesenchymal cells in the sheath cell layer, and formation of a fluid-filled lumen between the follicular cells. Extra-ovarian factors, such as gonadotropins and metabolic hormones, and intra-ovarian factors, such as angiogenic factors and steroid hormones, coordinate the precise development of the follicles (9–11). Estrogen is a key ovarian steroid hormone synthesized *via* a synergistic process occurring between theca cells and granulosa cells. It promotes follicular development by regulating the processes of angiogenesis, granulosa cell proliferation, and differentiation in the ovary (12–16). Estrogen is synthesized by transforming cholesterol into androgens (dehydroepiandrosterone, androstenediol, androstenedione, and testosterone) through a series of continuous modification processes, and then through the continuous hydroxylation reaction catalyzed by cytochrome P450 (CYP) enzyme (17). During this process, the CYP enzymes capture the electrons released by reduced nicotinamide adenine dinucleotide phosphate (NADPH) to support their catalytic activity (18, 19). Thus, the biosynthesis of estrogen involves a complex series of processes starting from cholesterol and involving a variety of enzymes.

The level and type of nutrition are some of the most important environmental factors that affect the reproductive performance of animals. It has been previously reported that the process of follicle development is influenced by diet (20–22). Seasonal estrus in yaks appears to be less obvious under the conditions of a lack of seasonal variation in food, suggesting that the process of follicular development in yaks is influenced by their nutritional status (3, 23). *N*-Carbamylglutamate (NCG) is an analog of *N*-acetylglutamate (NAG); both NCG and NAG can promote the synthesis of endogenous arginine and have been shown to promote the development of growing follicles in the chicken ovary (24). Preliminary findings of our previous study suggested that NCG promotes angiogenesis and cholesterol metabolism and positively regulates estrogen synthesis and ovarian development in yak ovaries (25). However, the metabolic mechanisms associated with estrogen synthesis in ovarian tissues during follicular development under the action of NCG have not been characterized.

Transcriptome sequencing is a well-established technology that is used to assess the changes in animals simultaneously and comprehensively in response to environmental and dietary changes at the transcriptional level. Metabolomic analysis can potentially help understand the ancillary metabolic changes induced by the process of post-transcriptional regulation, as it usually identifies the last step in the series of changes induced by external stimuli in living systems (26). Transcriptomic-coupled metabolomics can provide clues to understanding the metabolic pathways associated with the process of biosynthesis to explore the underlying molecular mechanisms. Thus, as a powerful tool, transcriptomics combined with metabolomics has been recently used to identify the gene regulatory networks and metabolic processes involved with the biosynthesis of steroid hormones in ovarian tissues during the process of follicular development (27–29). In this study, we conducted a comprehensive analysis of transcriptomic and metabolomic data of ovarian tissues to unveil the potential molecular mechanisms by which NCG manifests its effects on the process of follicle development in yaks. The results of the present study showed that dietary NCG supplementation increased the number of large follicles and estradiol levels in serum, and the integration of transcriptomic and metabolomic analysis indicated that this may be related to the pentose phosphate pathway.

## Materials and methods

### Approval by the ethics committee

We followed the guidelines outlined in the Guide for the Care and Use of Laboratory Animals developed by the National Research Council of China. All experimental protocols were approved by the Animal Ethical and Welfare Committee of Sichuan Agricultural University (#SCAUAC2020-84).

### Animals and experimental design

This study was carried out from August to October 2020 at the Dujiangyan Oujiapo Cattle Farm (30.59°N, 103.37°E), Chengdu, China. A total of twelve multiparity and non-pregnant female yaks (parity: 1.33 ± 0.49; body weight: 170.9 ± 28.83 kg; age: 4.67 ± 0.78 years) were randomly divided into two groups (Control and NCG) based on their weight, parity, and age parameters. The Control group was fed a basal diet and the NCG group was fed a basal diet supplemented with 6 g/day of NCG per yak, which based on the previous reports (30–33). After a 14-day adaptation period, the yaks were allotted individual pens and fed independently. All test yaks were fed a total mixed ration (TMR; the composition and nutrient composition are presented in Supplementary Table S1) at 7:00 a.m. and 5:00 p.m. NCG was temporarily and individually mixed in the TMR.

Progesterone vaginal suppositories were placed in the vagina of all yaks before the start of the formal trial (day 0) and extracted on day 12. Progesterone (1.38 g) was present in the vaginal suppositories, which were procured from Ningbo Sansheng Biological Technology Co., Ltd., Ningbo, China. The corpus luteum was lysed by intramuscularly injecting 2 mL of a commercial injection containing 0.4 mg of cloprostenol (Ningbo Sansheng) to induce estrus and ovulation, as per the manufacturer's instructions. The time of disappearance of estrus characteristics (day 16) was considered to be the beginning of the next ovarian cycle, and all yaks were slaughtered at the predicted time of the selection of the third-wave dominant follicles (day 32) in this follicular cycle (34).

### Sample collection

On days 28, 30, and 32, blood samples (5 mL) were collected from the jugular veins of yaks prior to morning feeding and separated at 3,000 rpm for 10 min to obtain serum. The serum sample was divided and stored at −20°C until further analysis. After slaughter, ovaries from each yak were isolated and washed using pre-cooled Dulbecco's phosphate-buffered saline (D-PBS, Gibco, Grand Island, NY, USA). Following this, the samples were cleaned using absorbent paper and then weighed. Visualized follicles (>1.0 mm in diameter) in the left ovary were classified following previously reported protocols (35) as 1.0–5.0 mm, 5.0–10.0 mm, or >10.0 mm, and the mean of each type of follicle was counted. Subsequently, the follicles were rapidly frozen in liquid nitrogen before being transferred to a −80°C refrigerator for storage.

### Serum hormone measurement

The serum levels of estradiol and progesterone were determined using commercial ELISA kits (#MM-247601 and #MM-5091801, MEIMIAN, Yancheng, China). The protocols outlined by the manufacturer were followed to conduct the process.

### Transcriptomic analysis

The ovarian samples (stored at −80°C) were ground to form powdered samples in liquid nitrogen. Following this, the total RNA was extracted using the RNA extraction and purification kit (#DP431, Tiangen, Beijing, China) according to the instructions provided by the manufacturer. The concentration of total RNA was determined using the NanoDrop 2000 spectrophotometer (Thermo Scientific, Wilmington. DE, USA) and the quality of the RNA samples was determined using the Agilent 2100 Bioanalyzer (Agilent Technologies, Pleasanton, CA, USA). The integrity of RNA was determined by ribonuclease-free agarose gel electrophoresis. Qualified RNA samples were sent to Gene Denovo Biotechnology Co. (Guangzhou, China) for transcriptome sequencing. The mRNA was purified and fragmented to synthesize cDNA. In the presence of random hexamer primers, the first strand of cDNA was synthesized using superscript II (Invitrogen, Carlsbad, CA, USA), and the second strand of cDNA was synthesized using DNA polymerase I and RNase H before end repair, data tracking, and adapter connection. The adapter modified fragments were then purified and amplified using the QiaQuick PCR Extraction Kit (Qiagen, Venlo, Netherlands) to create the final cDNA library. Six RNA-seq libraries were constructed and sequenced using the Illumina HiSeq 2500 platform (the Control and NCG groups consisted of three ovarian samples each). The Hisat2 version software was used to map the paired-end clean reads to the yak reference genome (36). The fragments per kilobase of transcript per million mapped reads (FPKM) were regarded as the gene expression levels. The differentially expressed genes (DEGs) were identified from the normalized read count data using the DESeq software (37). Genes characterized by fold change (FC) values > 1.5 or FC values < 0.66 and *p*-value < 0.05 were considered to be DEGs and were analyzed using the Gene Ontology (GO) database and Kyoto Encyclopedia of Genes and Genomes (KEGG) for further pathway enrichment analysis. The false discovery rate was used to adjust the *p*-value. An adjusted *p*-value (Q-value) of < 0.05 was considered as significant.

### Metabolomic analysis

Ovary samples (100 mg) ground in liquid nitrogen were placed in a centrifuge tube, an aqueous solution (500 μL) containing formic acid (0.1%, v/v) and methanol (80%, v/v) was added to it, and the mixture oscillated and vortexed. The resulting mixture was incubated on ice for 5 min and then centrifuged at 15,000 g for 10 min at 4°C. Water (mass spectrometry grade) was used to dilute the supernatant and the final concentration of methanol was 53%. The resulting solution was centrifuged at 15,000 g and 4°C for 10 min. Following this, the supernatant was collected, then transferred to a new Eppendorf tube and centrifuged at 15,000 g (temperature: 4°C; time: 20 min) (38). Finally, liquid chromatography-electrospray ionization tandem mass spectrometry (LC-MS/MS) was used to analyze the supernatant. A total of twelve samples (six ovarian samples belonging to the Control group and six samples belonging to the NCG group) were analyzed at Novogene Co., Ltd. (Beijing, China) using the Vanquish UHPLC system (Thermo Fisher, Germering, Germany), in combination with an Orbitrap Q Exactive™ HF mass spectrometer (Thermo Fisher). The Compound Discoverer 3.1 (CD3.1, ThermoFisher) was used to process the raw data files and execute the processes of peak alignment, peak pickup, and quantification of each metabolite. Partial least squares discriminant analysis (PLS-DA) and principal component analysis (PCA) were performed using the metaX software (comprehensive and flexible software that is used to process the metabolomics data) to assess grouping trends between treatments. We performed *t*-tests to calculate the statistically significant of the metabolites (39). Metabolites that simultaneously met the conditions of variable importance in the projection (VIP > 1), *p*-value < 0.05, and FC > 1.2 or FC < 0.833 were classified as differential metabolites (DMs) (40–42). The enriched KEGG pathways associated with the identified DMs were determined; the pathways were considered to be statistically significant enrichment at a *p*-value of < 0.05.

### Determination of the NADP^+^/NADPH ratio and glucose 6-phosphate dehydrogenase activity

The NADP^+^/NADPH ratio in the ovaries was determined using the NADP^+^/NADPH assay kit (#S0179, Beyotime, Shanghai, China), and the G6PDH activity in the ovaries was determined using the G6PDH activity assay kit (#BC0265, Solarbio, Beijing, China) according to the manufacturer's instructions. The activity of G6PDH was corrected for protein concentrations determined using the BCA protein assay kit (#P0012S, Beyotime) as per the manufacturer's instructions.

### Real-time polymerase chain reaction analysis

RT-PCR was used to determine the relative expression level of candidate genes. The ovarian RNA extracts were reverse-transcribed into cDNA using the PrimeScript™ RT kit (#RR037A, Takara, Tokyo, Japan). RT-PCR was conducted using the SYBR Premix Ex-TaqTM Kit (#Q431-02, Vazyme, Nanjing, China) and a QuantStudio™ 6 Real-Time PCR system (Applied Biosystems, Foster City, CA, USA). The comparative cycle threshold (2^−ΔΔCt^) method was used to determine the relative expression levels of the genes in the NCG group (relative to the expression levels of the Control group). The mean value of the Control yak ovary gene expression level was set at 1.00, and β-actin was considered to be the housekeeping gene. All gene sequences used in the process are listed in Supplementary Table S2.

### Analysis of data

We calculated Pearson correlation coefficients for the DMs and DEGs using the OmicShare tools (https://www.omicshare.com/tools) to investigate the potential regulatory network of NCG-regulated follicle development in yaks. The correlation between DMs and DEGs was considered significant at an absolute value of correlation coefficient |r| > 0.8 and *p*-value < 0.05. The DMs and DEGs were also mapped to the KEGG database to obtain their common pathway information and determine the main biochemical pathways and signal transduction pathways associated with the DEGs and DMs. The pathways associated with the metabolomes and transcriptomes were identified. Network interactions between metabolites and genes were studied using Novomagic, a free online platform for data analysis (https://magic.novogene.com). For data other than transcriptomic and metabolomic data, independent *t*-tests were conducted using the SPSS 26.0 software (SPSS Inc., Chicago, IL, USA) to analyze. The data were expressed as mean ± SEM. Each yak was considered to be an independent experimental unit. The significant differences were considered at *p* < 0.05.

## Results

### Development of follicles

**Figure 1:**
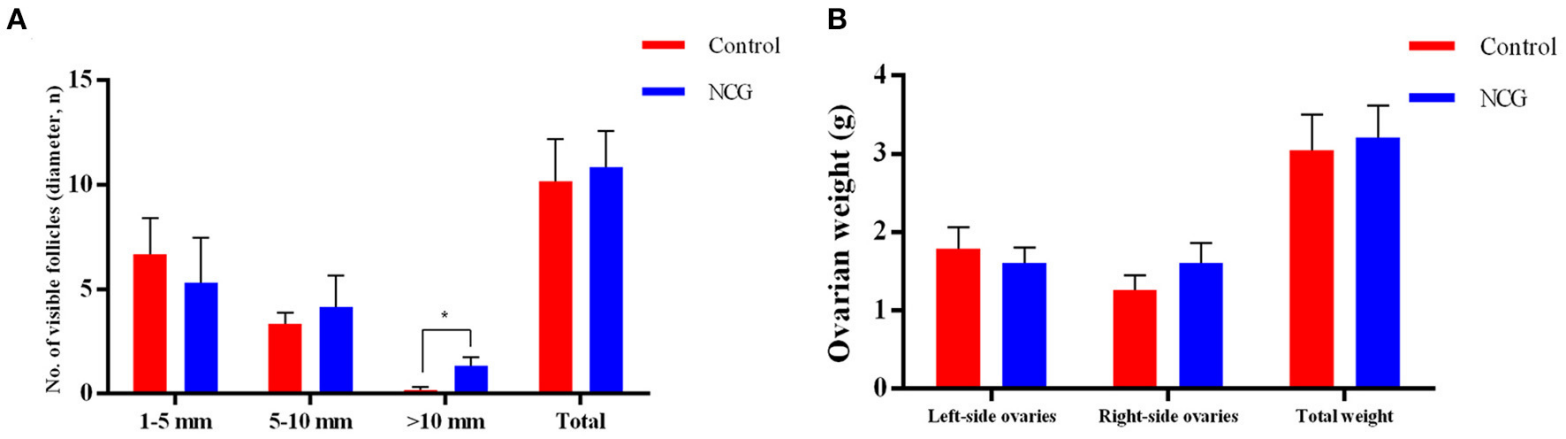
Average number of follicles of different sizes **(A)** and ovarian weight **(B)** for the two treatment conditions. All data are presented as mean and standard error. *P < 0.05.

To investigate the effect of NCG on follicle growth, the number of follicles under different classifications was assessed in both groups (Figure 1). In the presence of NCG, the number of follicles with >10.0 mm diameter increased significantly (*p* < 0.05). It was not observed that ovarian weight or the number of follicles with <10.0 mm diameter were affected by NCG. We found that NCG increased the number of large follicles.

### DEGs and transcriptome data analysis

**Figure 2:**
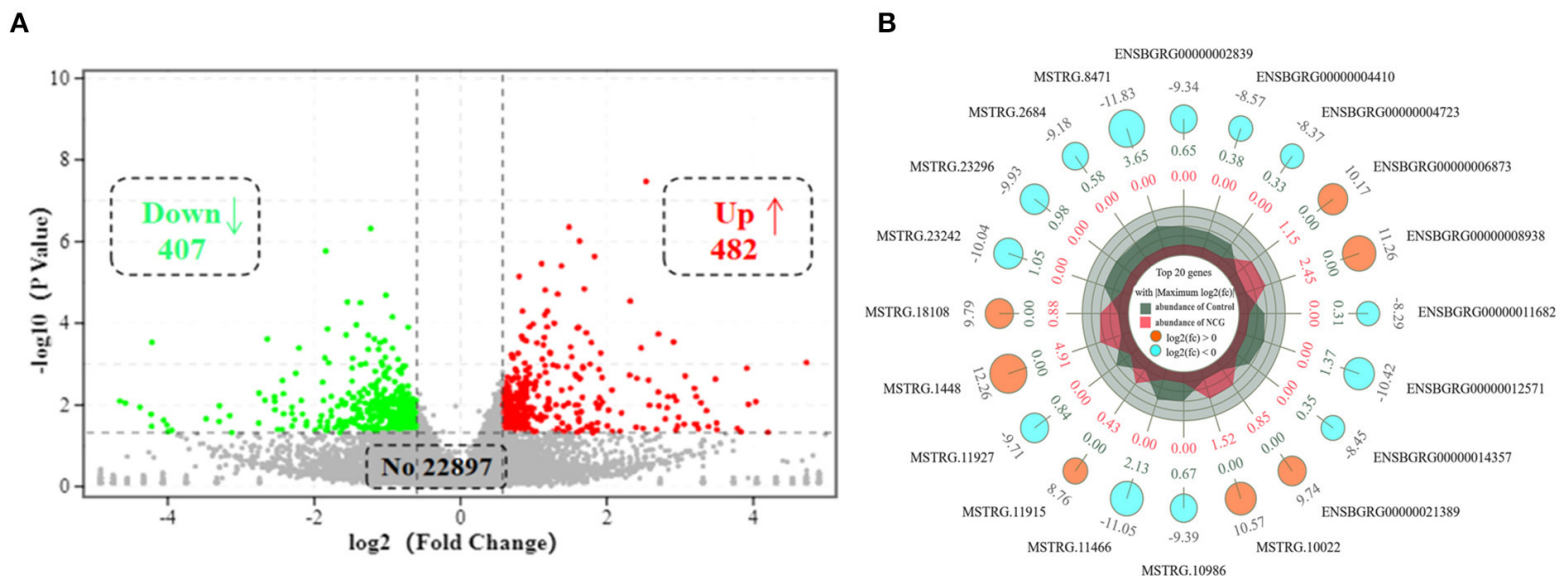
Volcano plot of **(A)** and top 20 **(B)** differentially expressed genes (DEGs) in yak ovaries for the Control and NCG groups.

To explore the effect of NCG on ovarian transcriptional profiles, RNA-seq as used to analyze the DEGs. After sequencing the cDNA library, the number of clean data in each library ranged from 42,625,084 to 45,966,826, and 93.87% of these reads were mapped to the yak reference genome. At least 93% of reads in each library scored Q30 or above, and the average GC content across all libraries was 50.40% (Supplementary Table S3). This indicated that the reads were of good quality and could be used for the analysis of the DEGs. A total of 889 DEGs (FC > 1.5 or FC < 0.666, and *p*-value < 0.05) were identified in ovarian tissues. Of the 889 DEGs, 407 were down-regulated, and 482 genes were up-regulated in the NCG group than in the Control group, as shown in Figure 2A. The top 20 genes with the maximum differences (selected with FC as a reference) are shown in Figure 2B.

**Figure 3:**
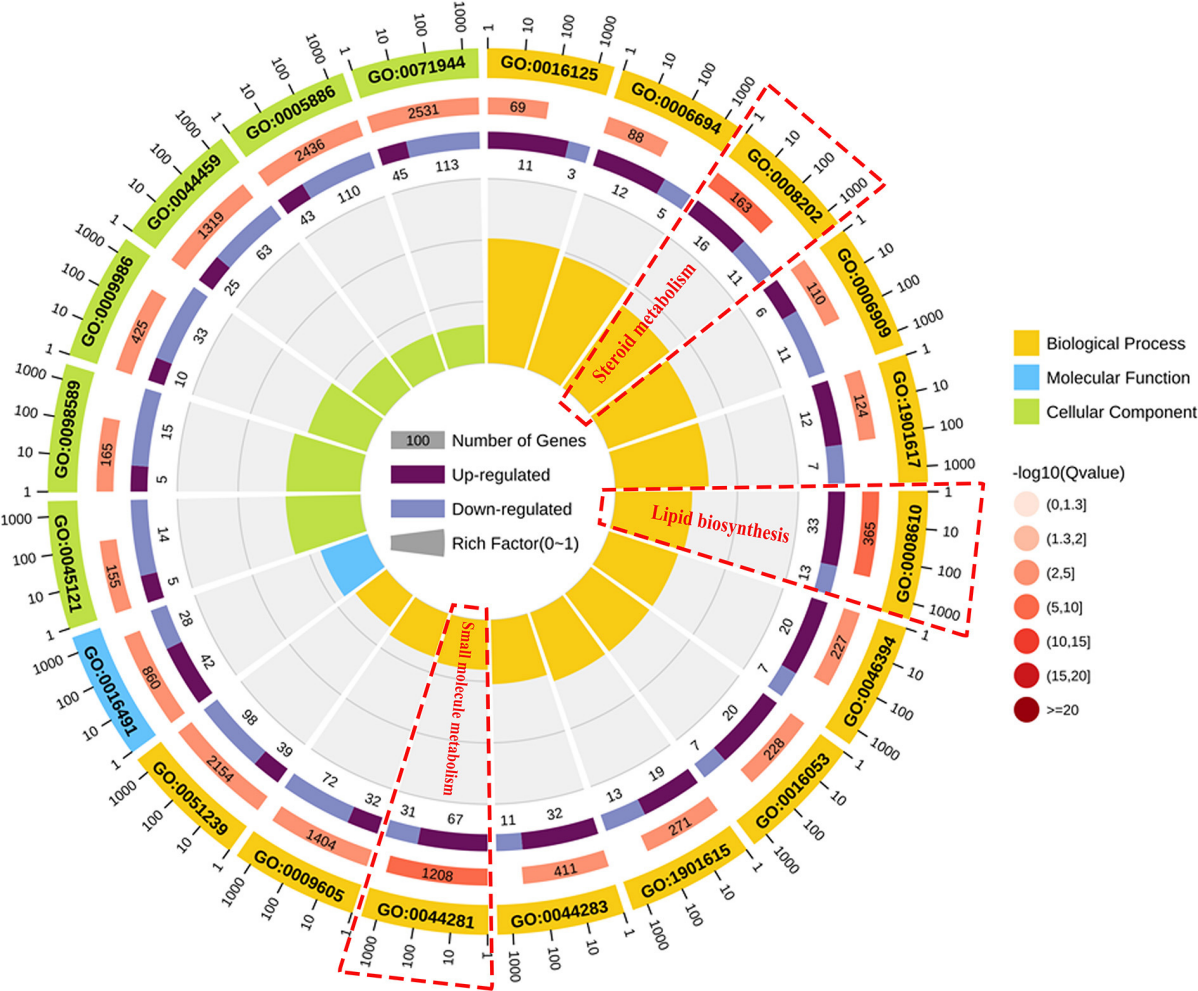
Function analysis of the DEGs between the control and NCG groups based on the results of the Gene Ontology (GO) analysis method. Top 20 terms (control vs. NCG) for GO enrichment. Yellow, blue, and green represent biological process, molecular function, and cellular component, respectively.

To classify the function of these DEGs, the functional categories of the identified DEGs were annotated following the GO enrichment analysis are shown in Supplementary Figure S1 and Supplementary Table S4. The DEGs were annotated into three categories, namely, biological process (BP), cellular component (CC), and molecular function (MF) composed of 26, 11, and 15 subcategories, respectively. Further, to obtain an overview of the effect of NCG on ovarian tissue, enrichment analysis of DEGs was performed based on the GO annotation terms. Of the 20 most significant GO terms, 13 belonged to the BP category, 6 to the CC category, and 1 to the MF category, as shown in Figure 3. This indicated that NCG primarily affected the metabolic processes of the ovarian tissues. Furthermore, the three show the most significant differences in the BP category (Q value < 0.05) were observed for the processes associated with lipid biosynthesis (GO: 0008610), steroid metabolism (GO: 0008202), and small molecule metabolism (GO: 0044281). We listed the rich interaction network of GO terms associated with BP, CC, and MF to assess the interrelationship between the DEGs (Supplementary Figures S2–S4).

**Figure 4:**
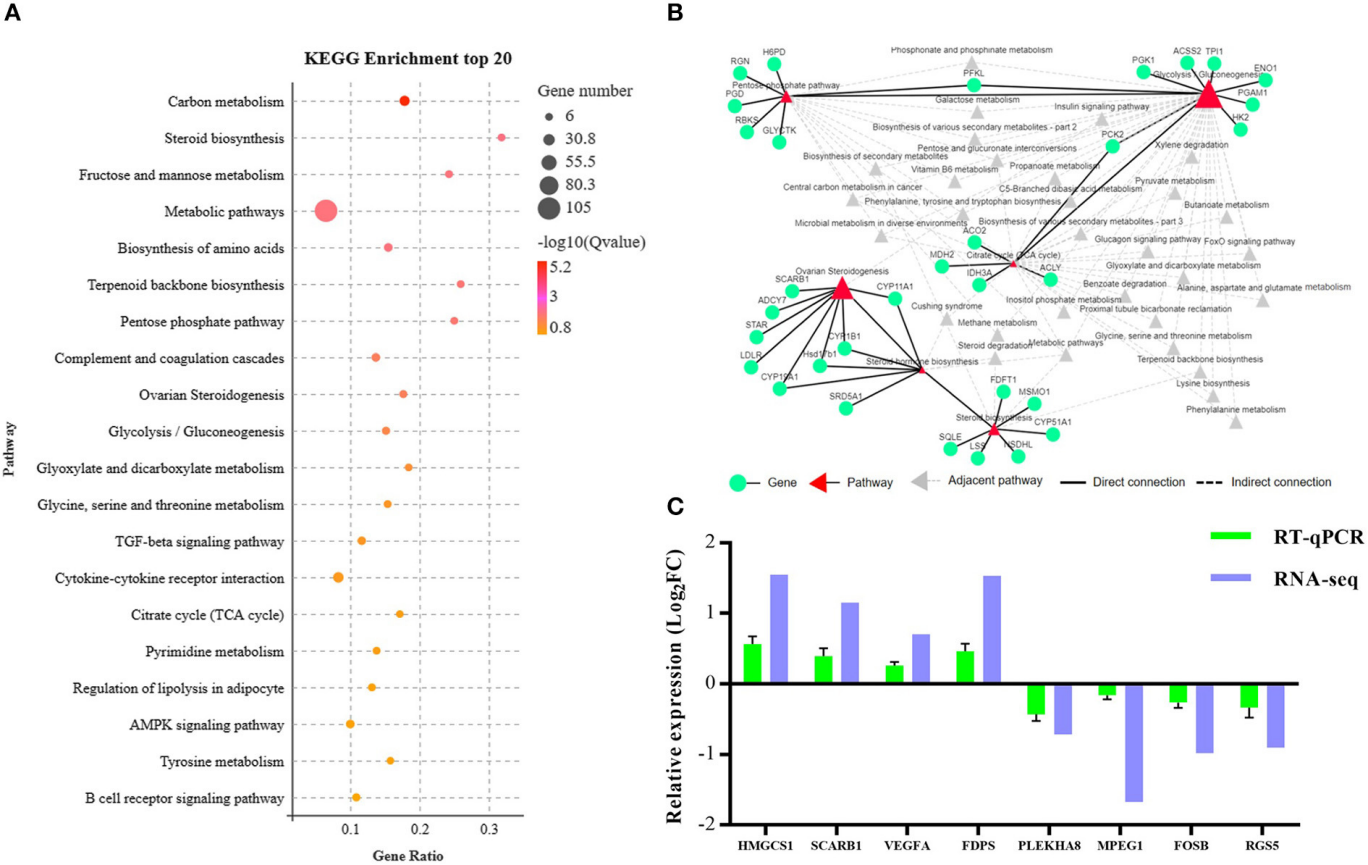
Bioinformatics analysis and validation of DEGs between the NCG and Control groups based on the data presented in the Kyoto Encyclopedia of Genes and Genomes (KEGG) pathway analysis. **(A)** Top 20 terms (Control vs. NCG) for KEGG enrichment; **(B)** Correlation network corresponding to the DEG enriched pathways associated with steroid biosynthesis, pentose phosphate pathway, ovarian steroidogenesis, glycolysis/gluconeogenesis, and citrate cycle (TCA); **(C)** Expression levels of the eight functional DEGs as determined by RNA-seq using RT-PCR, *n* = 6.

To systematically analyze the functions of DEGs, KEGG enrichment analysis was used to identify the different pathways associated with the identified DEGs. We found that DEGs were primarily involved with lipid metabolism, carbohydrate metabolism, metabolism of terpenoids and polyketides, endocrine system, etc. (Supplementary Table S5). Furthermore, these DEGs were enriched in steroid biosynthesis, pentose phosphate pathway, ovarian steroidogenesis, glycolysis/gluconeogenesis, tricarboxylic acid cycle (TCA cycle), and other pathways as presented in Figure 4A. We established the network interactions among these pathways and the DEGs associated with different pathways to explore the connections between these enriched pathways (Figure 4B).

To validate the RNA-Seq data, eight genes (3-hydroxy-3-methylglutaryl-CoA synthase 1, *HMGCS1*; scavenger receptor class B member 1, *SCARB1;* vascular endothelial growth factor A, *VEGFA;* farnesyl diphosphate synthase, *FDPS;* pleckstrin homology domain containing A8, *PLEKHA8;* macrophage expressed 1, *MPEG1;* fosB proto-oncogene, *FOSB;* regulator of G protein signaling 5, *RGS5*) were randomly selected from the list of functional DEGs for validation by RT-PCR, and the results were consistent with that of RNA-seq analysis (Figure 4C).

### Analysis of DMs and metabolomic data

To explore the changes in metabolic profile of yak ovaries following the addition of NCG into the diet, LC-MS/MS were performed. Multivariate statistical analysis was used to analyze the identified compounds. PCA was used to study the general distribution trend of the metabolites in the two groups of samples. The PLS-DA method was used to model the relationship between the expression levels of the metabolites and the sample class to enable the prediction of the sample class. As shown in Supplementary Figure S5, the PCA revealed that the samples supplemented with or without NCG formed separate clusters. The six biological replicates in the same group were grouped in the same cluster, indicating that a large difference existed between the two groups. The Orthogonal projections to latent structures discriminant analysis (OPLS-DA) revealed that the NCG and Control groups could be separated along the x-axis (Supplementary Figure S6). These results of PCA and PLS-DA revealed that the addition of NCG to the diet resulted in a significant alteration of the ovarian metabolic profile in yaks.

**Figure 5:**
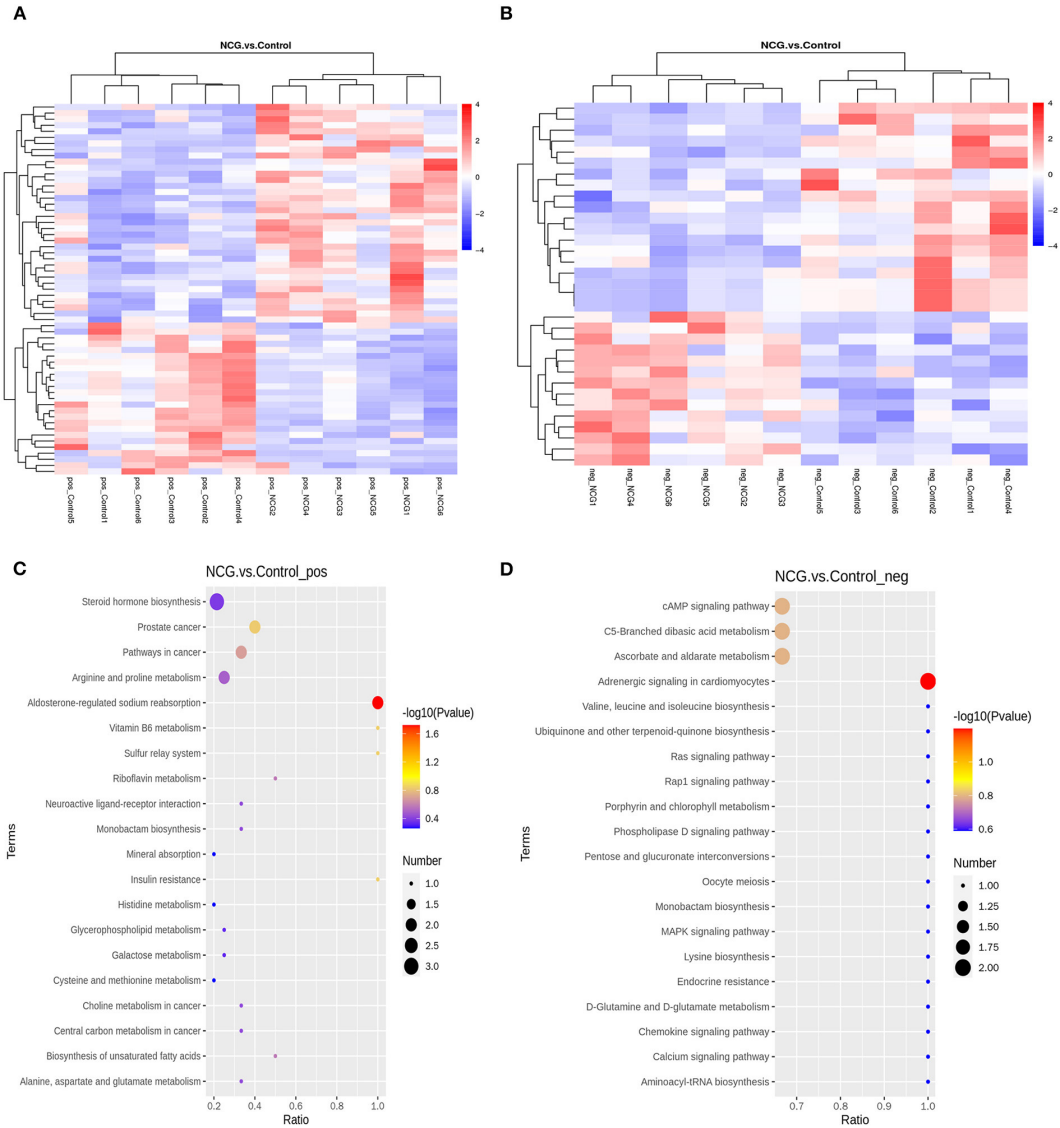
Cluster heatmap for differential metabolites (DMs) under the positive **(A)** and negative ion modes **(B)** for the two treatment groups. Diagrams indicating the KEGG pathway enrichment degree for the DMs in the positive **(C)** and negative ion modes **(D)**.

A total of 94 DMs with VIP > 1, *p*-value < 0.05, FC > 1.2 or FC < 0.833 of the 622 metabolites were identified. Under the positive ion mode, of the 393 metabolites, we identified 61 DMs, 36 of which were up-regulated and 25 were down-regulated in the NCG group (Supplementary Table S6). Under the negative ion mode, of the 229 metabolites, we identified 33 DMs, 14 of which were up-regulated and 19 were down-regulated in the NCG group (Supplementary Table S7). Figures 5A,B present the expression patterns of the DMs. The KEGG enrichment analysis method was used to understand the biological mechanisms associated with the process of follicle development. DMs in the positive analysis ion mode were annotated to be associated with 24 pathways (Supplementary Table S8), and DMs in the negative analysis ion mode were annotated to be associated with 79 pathways (Supplementary Table S9). The pathways included the pentose and glucuronate interconversion pathways, pathways associated with the TCA cycle, and the pathways associated with the steroid hormone biosynthesis. The top 20 of the most significant pathways in the positive and negative ion modes are listed in Figures 5C,D.

### Integrated analysis of the transcriptomic and metabolomic data

**Figure 6:**
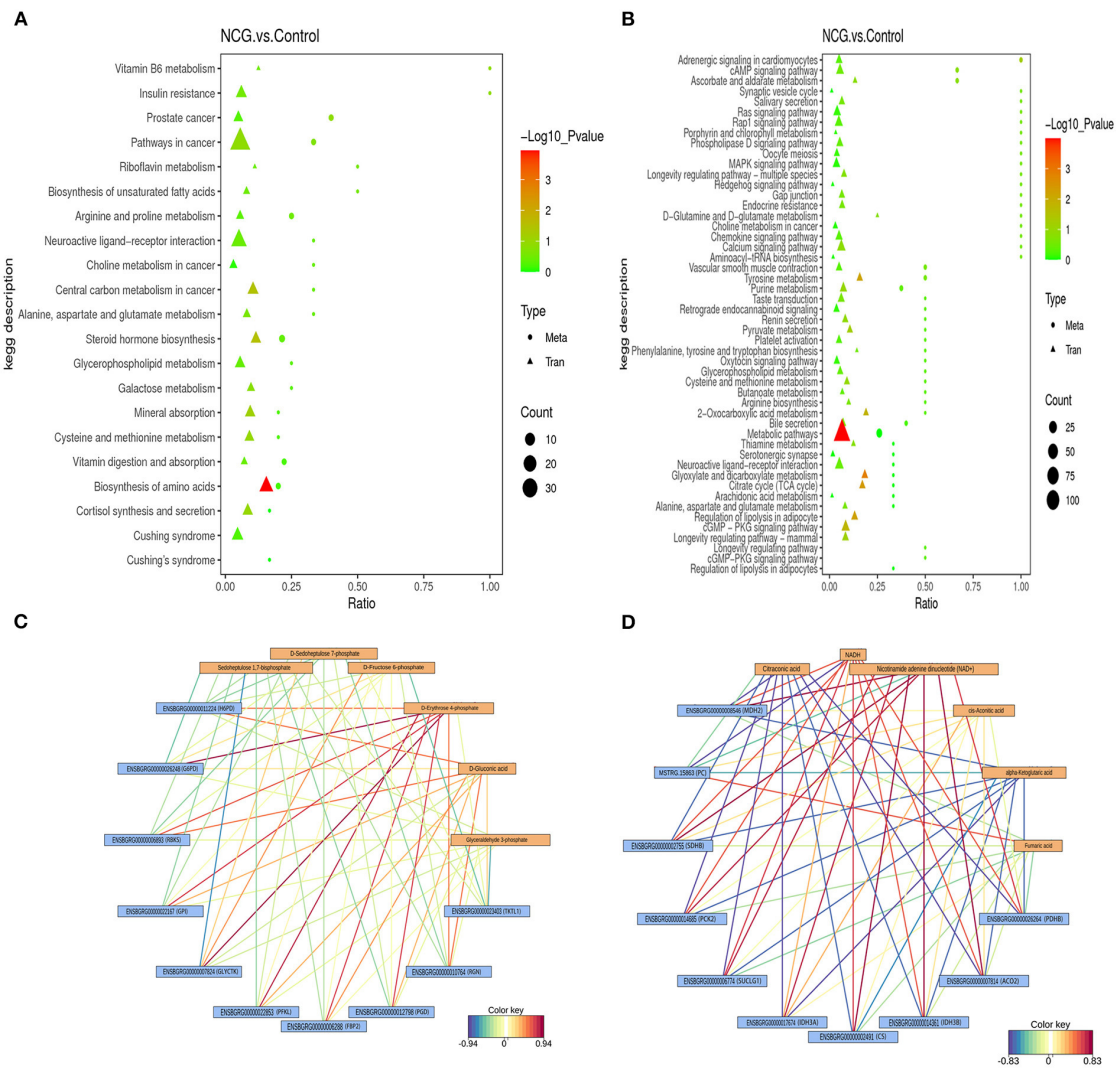
Integrated altered metabolic pathways identified based on the transcriptomic and metabolomic data. The vertical axis presents the pathway description under the positive **(A)** and negative **(B)** ion modes, and the horizontal axis presents the ratio of the DEGs and DMs. Correlation network corresponding to DEG- and MD-enriched pathways associated with the pentose phosphate pathway **(C)** and TCA cycle **(D)**.

To explore the potential relationship between the DEGs and DMs by integrating the date of transcriptome and metabolome at the pathway level. Twenty-one enrichment pathways were identified under the positive ion mode (Figure 6A). These pathways included those associated with the processes of vitamin B6 metabolism, biosynthesis of unsaturated fatty acids, galactose metabolism, steroid hormone biosynthesis, biosynthesis of amino acids, etc. As shown in Figure 6B, 50 enrichment pathways, including the cAMP signaling pathway, MAPK signaling pathway, pathways associated with pyruvate metabolism, pathways associated with the TCA cycle, pathways associated with arginine biosynthesis, and 2-oxocarboxylic acid metabolism pathways among others, were identified under the negative analysis ion mode. The processes of energy metabolism and steroid hormone synthesis play an important role in the potential relationship between the DEGs and DMs. Therefore, the results suggest that the NCG-regulation of follicle development in yak can be potentially associated with the processes associated with steroid hormone synthesis and energy metabolism. A heat map of integration analysis corresponding to the expression patterns of the DEGs and MDs is presented in Supplementary Figure S7. The pentose phosphate pathway and TCA cycle were enriched by both DEGs and DMs (KEGG database), and the correlation network maps corresponding to the DEGs and DMs associated with these two pathways are listed in Figures 6C,D. The levels of D-erythrose 4-phosphate and D-gluconic acid were positively correlated with the gene expression of almost all DEGs associated with the pentose phosphate pathway. The levels of both two chemical forms of icotinamide adenine dinucleotide (NADH and NAD^+^) were positively correlated with the gene expression of almost all DEGs associated with the TCA cycle pathway, while the levels of citraconic acid and α-ketoglutaric acid were negatively correlated with the gene expression of almost all DEGs associated with the TCA cycle pathway.

### Serum hormone

To investigate the cause of follicle growth in response to NCG, estrogen levels in serum were examined (Table 1). The concentration of serum estradiol in the NCG group was higher than that in the Control group on days 28, 30, and 32 (*p* < 0.05), whereas the concentration of progesterone in the NCG group was lower than that in the Control group at day 30 (*p* = 0.025). Supplementation of NCG increased the serum levels of estradiol.

**Table 1:** Levels of serum estradiol and progesterone on days 28, 30, and 32.

**Item**	**Control**	**NCG**	**SEM**	***p*-value**
**Day 28**
Estradiol (pg/mL)	41.32	54.25	2.81	0.012
Progesterone (ng/mL)	0.70	0.61	0.05	0.335
**Day 30**
Estradiol (pg/mL)	42.06	53.13	2.23	0.005
Progesterone (ng/mL)	0.98	0.76	0.05	0.025
**Day 32**
Estradiol (pg/mL)	44.18	52.95	2.21	0.040
Progesterone (ng/mL)	1.01	0.86	0.05	0.131

### NADP^+^/NADPH ratio and glucose 6-phosphate dehydrogenase activity in the ovaries

**Figure 7:**
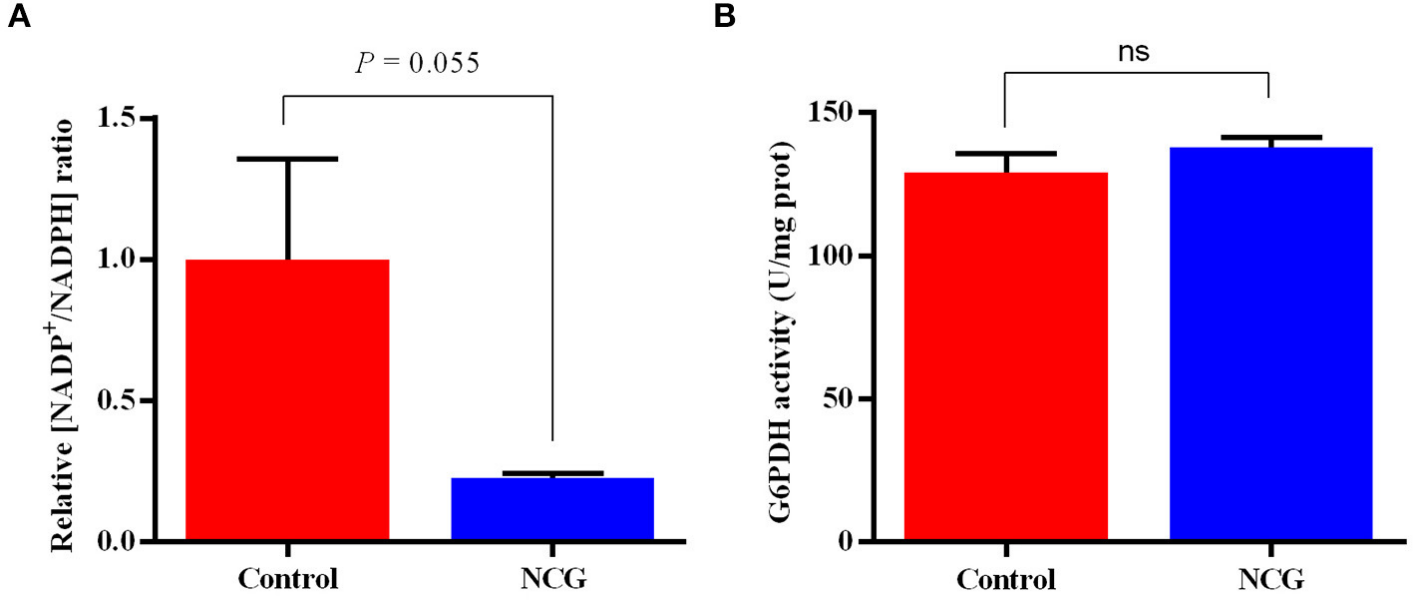
Relative NADP+/NADPH ratio **(A)** and G6PDH activity **(B)** in the ovaries of the control and NCG groups, *n* = 6.

To validate the pentose phosphate pathway, key enzymes and products in this pathway were examined (Figure 7). The NADP^+^/NADPH ratio in the ovaries of the NCG group was lower than that in the control group (*p* = 0.055). There was no difference in G6PDH activity between the two groups. It was observed that NCG supplementation had no significant effect on the key enzyme activities and products of pentose phosphate pathway.

### Gene expression related to steroid hormone biosynthesis and pentose phosphate pathway

**Figure 8:**
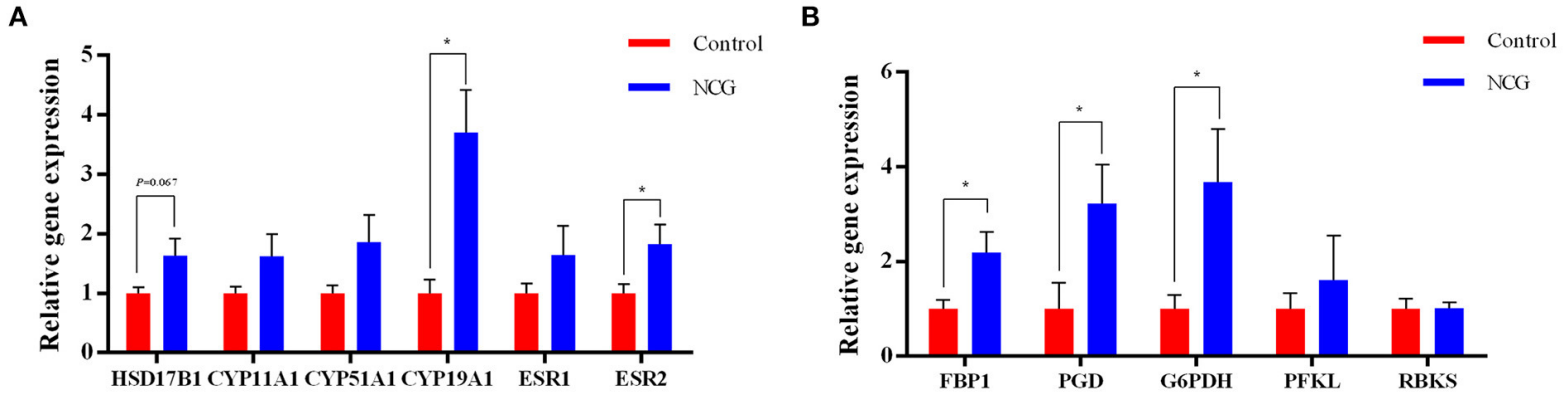
Relative gene expression of the genes associated with steroid biosynthesis **(A)** and the pentose phosphate pathway **(B)** determined using RT-PCR. The data are presented as the mean and standard error, *n* = 6, *P < 0.05.

To validate steroid hormone synthesis and the pentose phosphate pathway, the expression of key genes in these two pathways were examined (Figure 8). The relative expression of *CYP19A1* gene encoding aromatase, *ESR2* encoding estrogen receptor 2, *FBP1* encoding fructose-bisphosphatase 1, *PGD* encoding phosphogluconate dehydrogenase, and *G6PDH* encoding glucose-6-phosphate dehydrogenase were significantly up-regulated in the NCG group (*p* < 0.05) compared with the Control group. Furthermore, the relative gene expression of *HSD17B1* encoding hydroxysteroid 17-beta dehydrogenase 1 was up-regulated in the NCG group (*p* = 0.067) compared with the Control group. Supplementation of NCG upregulated the mRNA levels of related genes in the estrogen synthesis and pentose phosphate pathways.

## Discussion

In most bovine ovarian cycles, dozens of small follicles are activated and develop synchronously within two to three cycles; this phenomenon is referred to as a follicular wave (34). During the process of follicular development, the largest follicle in each follicular wave that can continue to grow is defined as the dominant follicle (43, 44). The dominant follicles in the first follicular wave (days 8–11 of the ovarian cycle) develop at the same time as the corpus luteum during the first half of the ovarian cycle and thus, become anovulatory follicles and undergo atresia (45, 46). The dominant follicle is re-selected in the second or third follicle wave (days 9–16) and the dominant follicle proceeds to become an ovulatory follicle, while the remaining follicles in the same follicle wave degenerate due to atresia (47). In yaks, the serum estrogen levels gradually increase during the late luteal phase (days 13–19 of the ovarian cycle) (23). Therefore, we chose to collect ovaries for analysis at the time after the completion of the second follicular wave of the ovarian cycle.

During the evolution of the follicle (transformation from the primordial state to the ovulatory stage), the ovarian follicle cells undergo extensive proliferation and differentiation. In this process, various steroid hormones in the follicular fluid (synthesized by ovarian tissues or obtained from the diet and environment) influence the follicular growth and development process. Different steroid hormones affect the intrafollicular environment by regulating cell proliferation, angiogenesis, and apoptosis of the follicle cells (11). Estradiol, the major and biologically active estrogen synthesized and secreted by growing follicle cells, is a key component of the physiological cascade response that stimulates estrous behavior and induces gonadotropin surge (48). Some of the paracrine or autocrine effects of estradiol in the ovary include an increase in ovarian weight, stimulation of ovarian angiogenesis, promotion of the process of follicular granulosa cell proliferation, increase in the sensitivity of the dominant follicle toward follicle-stimulating hormone, and decrease in the apoptosis of follicle cells (11). These factors orchestrate orderly follicular maturation. The concentration of estradiol in follicular fluid correlates positively with the volume of follicles (11, 49–52). This can be attributed to an increased number of granulosa cells synthesizing estradiol in the large follicles, as well as the greater mRNA abundance of genes encoding steroidogenic enzymes in large follicles (53). Therefore, the concentration of serum estradiol is considered a marker of follicular status, while the follicular diameter is an indicator of follicular growth and maturation (54, 55). In this study, we observed that dietary supplementation with NCG significantly increased the number of large follicles (diameter > 10.0 mm). This may be attributed to the increase in estrogen synthesis. It has been previously reported that NCG, an activator of carbamoyl phosphate synthase-1 and pyrroline-5 carboxylate synthase in the ornithine cycle, increases the synthesis of endogenous arginine (56) and promotes the process of ovarian tissue angiogenesis to provide adequate nutrients and oxygen, and favorable conditions for follicle growth and development (24, 25). Dietary supplementation with NCG has also been reported to increase blood estradiol concentrations in yaks (25) and chickens (57). Supplementation of NCG also promoted cell proliferation and inhibited apoptosis of pig placental trophoblast cells (58), and stimulated cell proliferation of bovine granulosa cells (59), both of which can synthesize estrogen. However, the precise molecular mechanism underlying this phenomenon has not been unveiled. Integrated transcriptomic and metabolomic techniques can be used to further elucidate the metabolic pathways associated with NCG regulation of estradiol synthesis at the molecular level.

The process of estradiol synthesis is initiated when cholesterol is transported from the outer mitochondrial membrane of theca cell to the mitochondrial inner membrane, where cholesterol is converted to pregnenolone under the action of the CYP cholesterol side-chain cleavage complex (CYP11A1). Subsequently, pregnenolone is sequentially converted to progesterone and androstenedione catalyzed by 3β-hydroxysteroid dehydrogenase (3BHSD) and CYP 17α-hydroxylase (CYP17A1). Progesterone and androstenedione then diffuse through the basement membrane to the granulosa cells where androstenedione is converted to testosterone under the catalytic action of hydroxysteroid 17-β dehydrogenase (HSD17B) and further catalyzed by CYP19A1 to estradiol. Estradiol is released into the follicular fluid and enters the blood circulation. In the biosynthesis of estradiol, the transcript levels of CYP11A1 and the activities of CYP17A1 and CYP19A1 are considered the controllers of the rate of estradiol synthesis (17, 60, 61). The CYP enzymes involved in estradiol synthesis are all oxidases containing an artificial heme group, whose catalytic activity requires electrons provided by NADPH (62). The function of hydroxysteroid dehydrogenase in steroid hormone synthesis also needs NADPH as a cofactor to provide electrons (63). Therefore, estrogen biosynthesis is limited by the availability of NADPH, which determines the efficiency of the catalyzing enzymes in this process.

In this study, we analyzed transcriptomics and metabolomics data and found that NCG not only affected the biosynthesis of steroid hormones but also regulated the TCA cycle and pentose phosphate pathway. More specifically, the expression of genes, namely, phosphofructokinase (*PFKP*), *G6PDH*, fructose-bisphosphatase 2 (*FBP2*), *PGD*, and ribokinase (*RBKS*), and the levels of metabolites, namely, -phosphogluconic acid, sedoheptulose 1,7-bisphosphate, and fructose 6-phosphate (F6P) of the pentose phosphate pathway in the NCG group was significantly higher than those in the control group. To verify these results, we examined the activity and relative expression level of *G6PDH*, a key enzyme associated with the pentose phosphate pathway in ovaries. We found that the activity of G6PDH in the NCG and control groups were not significantly different from each other, while the gene expression of *G6PDH* was significantly up-regulated in the NCG group (compared to the Control group). The pentose phosphate pathway is an important component of the glucose metabolism process where glucose 6-phosphate (G6P) is consumed, and glyceraldehyde 3-phosphate (G3P) and F6P are generated *via* the non-oxidative and oxidative branches of the pentose phosphate pathway, respectively. Unlike other glucose metabolic pathways (aerobic oxidation and glycolysis), the pentose phosphate pathway generates NADPH rather than adenosine triphosphate (ATP). It provides necessary electrons for the synthesis of fatty acids, sterols, nucleotides, and non-essential amino acids (64, 65). The rate-limiting enzyme of the oxidized pentose phosphate pathway is G6PDH, which dictates the flow rate of G6P into this pathway. It converts nicotinamide adenine dinucleotide phosphate (NADP^+^) to NADPH during the conversion of G6P to 6-phosphogluconolactone. In mammals, the pentose oxidative phosphate pathway is considered the major contributor to NADPH (66). In this study, the higher expression of *G6PDH* and the lower ratio of NADP^+^/NADPH in the NCG group indicated that NCG supplementation contributed to the activation of pentose phosphate pathway. Abnormalities in the pentose phosphate pathway hinder the process of oocyte maturation. The hindrance can be attributed to purine substrate deficiency and reduced NADPH production, and it can potentially affect the TCA cycle (67). Inhibition of the pentose phosphate pathway following the administration of dehydroepiandrosterone (DHEA, a known non-competitive inhibitor of G6PDH) to mice resulted in reduced ovulation rates, disruption of the motility cycle, and altered hormone levels (67, 68). In an *in vitro* assay, DHEA-treated blastocyst cells of porcine showed a higher apoptosis rate and a significantly lower cell proliferation rate, with lower NADPH levels in oocytes (69). In addition to providing NADPH to initiate the catalytic activity of CYPs for estrogen synthesis, the pentose phosphate pathway has positive roles in alleviating oocyte aging and oxidative status (70). Therefore, the regulation of yak follicle development by NCG may be associated with the improvement in the efficiency of the pentose phosphate pathway.

Interestingly, we found that the levels of the intermediates of the TCA cycle such as: citraconic acid, alpha-ketoglutaric acid, and isocitric acid in the NCG group were lower than that in the Control group. However, the expression of genes encoding metabolic catalytic enzymes, such as citrate synthase (*CS*), aconitase (*ACO*), malate dehydrogenase (*MDH*), ATP citrate lyase (*ACLY*), and pyruvate carboxylase (*PC*), in the TCA cycle was significantly higher in the NCG group than in the Control group. Previous studies have shown that NCG could improve the activity of the key enzymes associated with the TCA cycle in the intestine and liver of intrauterine growth-restricted fetal lambs (71, 72). Besides the pentose phosphate pathway, glucose produces pyruvate *via* both the aerobic oxidative pathway and the anaerobic glycolytic pathway, and pyruvate is the major energy source for oocytes (73). Glucose is oxidatively dehydrogenated and decarboxylated directly in the pentose phosphate pathway. During this process, it does not produce pyruvate for the subsequent TCA cycle. Thus, potentiation of the pentose phosphate pathway has the potential to inhibit the progression of the TCA cycle. The first step of the TCA is the condensation of acetyl coenzyme A (acetyl CoA) with oxaloacetate under the action of citrate synthase to form citrate. Acetyl CoA, as a precursor of cholesterol, is also involved in the synthesis of steroid hormones in the ovary. It has been reported that NCG could regulate cholesterol metabolism in the yak ovaries, thereby promoting estrogen synthesis (25). Since acetyl CoA is synthesized to cholesterol, less acetyl CoA enters the TCA cycle. Although inhibition of the TCA cycle significantly inhibits the growth of the follicles in mice (*in vitro*) (74), direct evidence of the association between the TCA cycle and the follicle development process is lacking and must be investigated further.

This study is limited in that we studied the ovarian tissue as a whole, which is rich in vascular, epithelial, and connective tissues besides follicles. This limits the accuracy of our results regarding the mechanism by which NCG regulates follicle development. Moreover, we explored the possible mechanism of the action of NCG in the regulation of yak follicle development, while the effect on yak fecundity remains unexplored and must be further confirmed by the conception rate and calving rate.

To the best of our knowledge, this study is the first to combine metabolomic and transcriptomic data to provide insights into the regulatory mechanisms associated with NCG for follicular development in yaks. The transcriptomic data revealed that the addition of NCG to the diet induced significant changes in the expression of several genes involved with lipid metabolism, carbohydrate metabolism, metabolism of terpenoids and polyketides, and the endocrine system. The changes in the metabolic profiles are usually consistent with the transcriptomic results, however, the transcriptome was much more variable. Analysis of the gene metabolite network revealed that NCG regulates the pentose phosphate pathway, TCA cycle, and steroid hormone biosynthesis pathway in the ovary of yaks. Our findings suggest the basic mechanisms by which NCG promotes follicular development in animals. Furthermore, our findings provide useful guidelines for and promote the use of NCG and other nutrients for the regulation of the reproductive performance of animals.

## Data availability statement

The datasets generated for this study can be found in online repositories. The raw sequence data obtained were deposited at NIH's Sequence Read Archive (SRA) under the accession number SRR14860524, SRR14860523, SRR14860522, SRR14860521, SRR14860520, and SRR14860525 (https://www.ncbi.nlm.nih.gov/bioproject/PRJNA738716).

## Ethics statement

The animal study was reviewed and approved by the Animal Ethical and Welfare Committee of Sichuan Agricultural University (#SCAUAC2020-84).

## Author contributions

BaX and JZ designed the study. JZ and JD performed the animal and laboratory experiments. JZ, SY, BeX, LW, QP, HZ, YJ, and RH provided laboratory experimental method and analyzed the data. JZ and SY wrote the original manuscript. BaX and ZW revised the final manuscript. All authors contributed to the article and approved the submitted version.

## Funding

This study was supported by the National Key R&D Program of China (2021YFD1600202 and 2018YFD0502303).

## Conflict of interest

The authors declare that the research was conducted in the absence of any commercial or financial relationships that could be construed as a potential conflict of interest.

## Publisher's note

All claims expressed in this article are solely those of the authors and do not necessarily represent those of their affiliated organizations, or those of the publisher, the editors and the reviewers. Any product that may be evaluated in this article, or claim that may be made by its manufacturer, is not guaranteed or endorsed by the publisher.
