# Supplementary material for: Integration of transcriptomic and metabolomic analysis of the mechanism of dietary N-carbamoylglutamate in promoting follicle development in yaks
Source: Front Vet Sci. 2022 Aug 29;9:946893. doi: 10.3389/fvets.2022.946893 (PMC9464987; doi:10.3389/fvets.2022.946893)
Supplement: Supplementary file 1 [file Data_Sheet_1.doc]

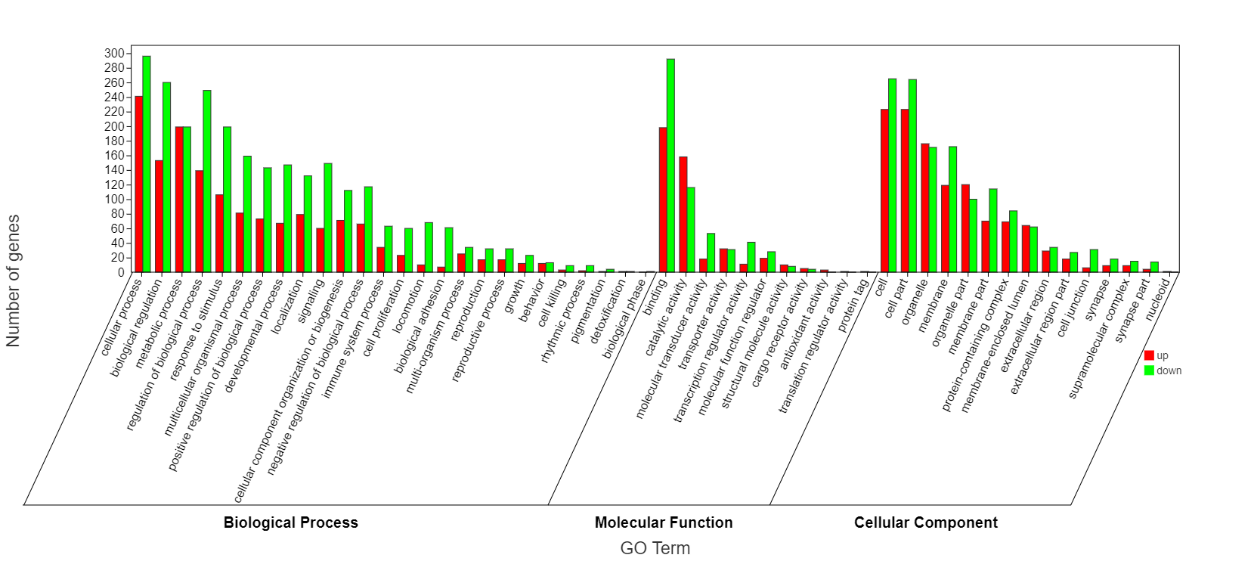


Figure S1. Gene ontology (GO) enrichment analysis. The genes from hierarchical clustering are further analyzed by using the database to realize annotation visualization and integrated discovery. The abscissa indicates the functions of GO analysis; the ordinate indicates the numbers of differentially expressed genes annotated.


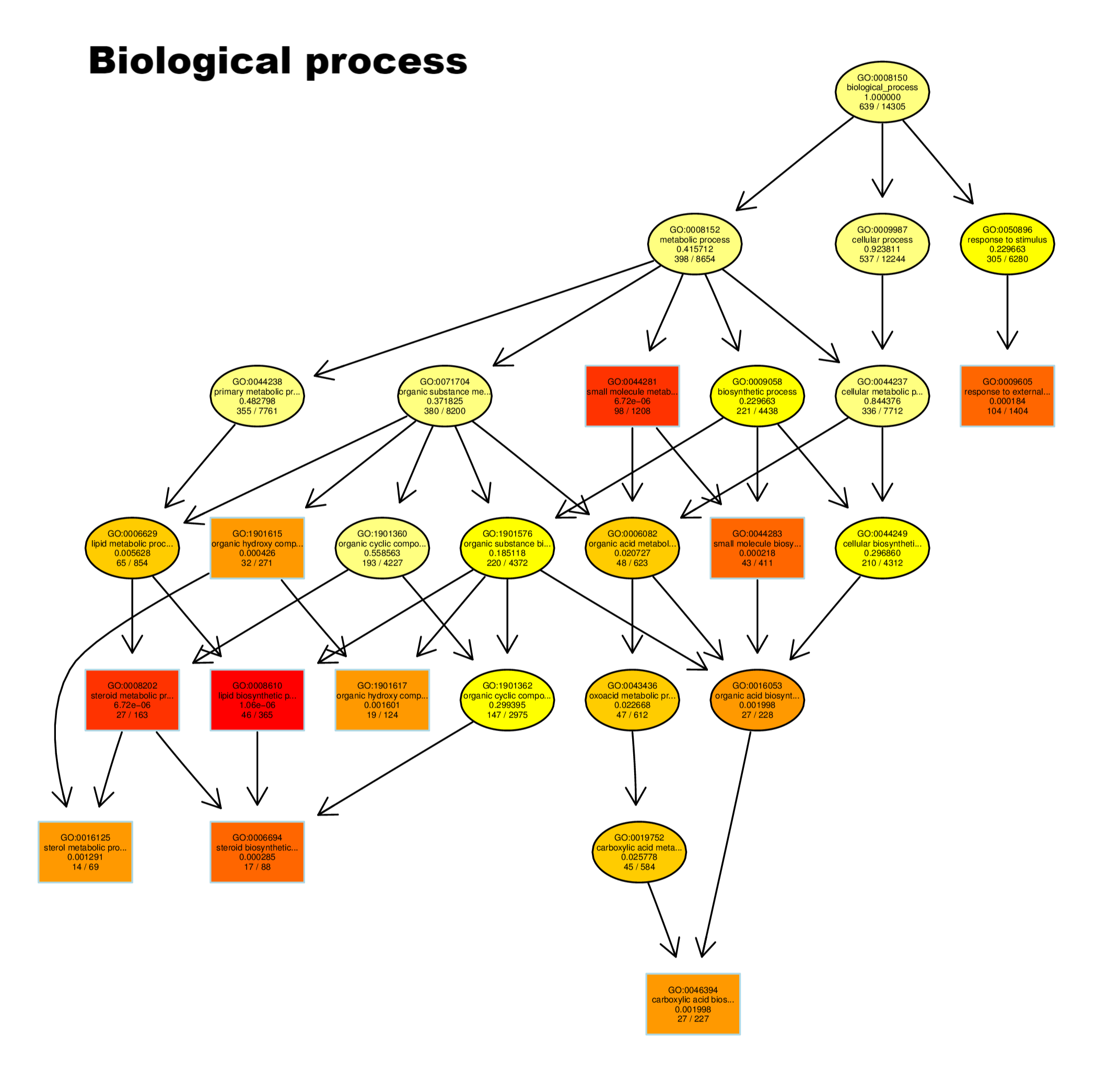
Figure S2. Interaction network corresponding to the enriched GO terms in biological process.


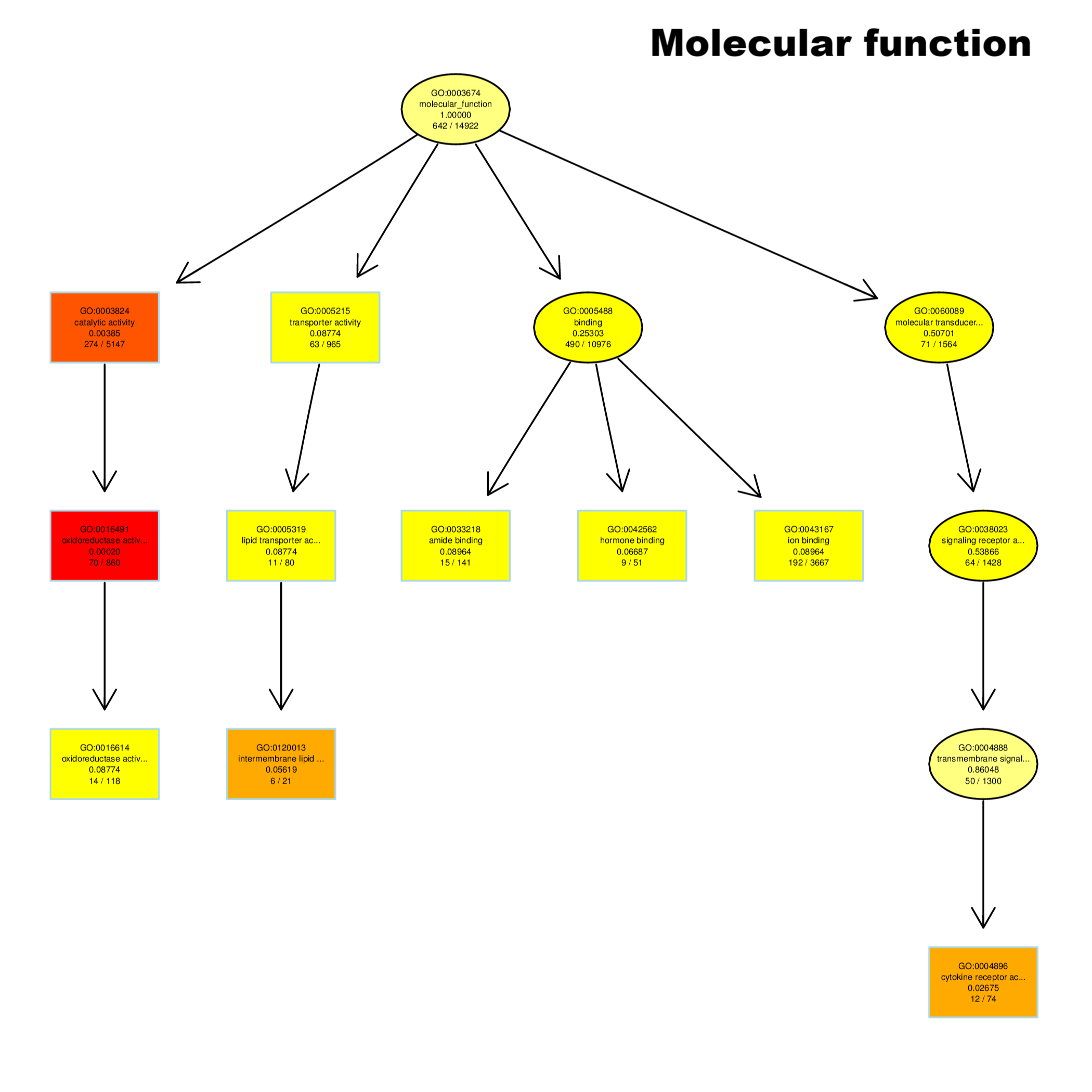
 Figure S3. Interaction network corresponding to the enriched GO terms in molecular function.
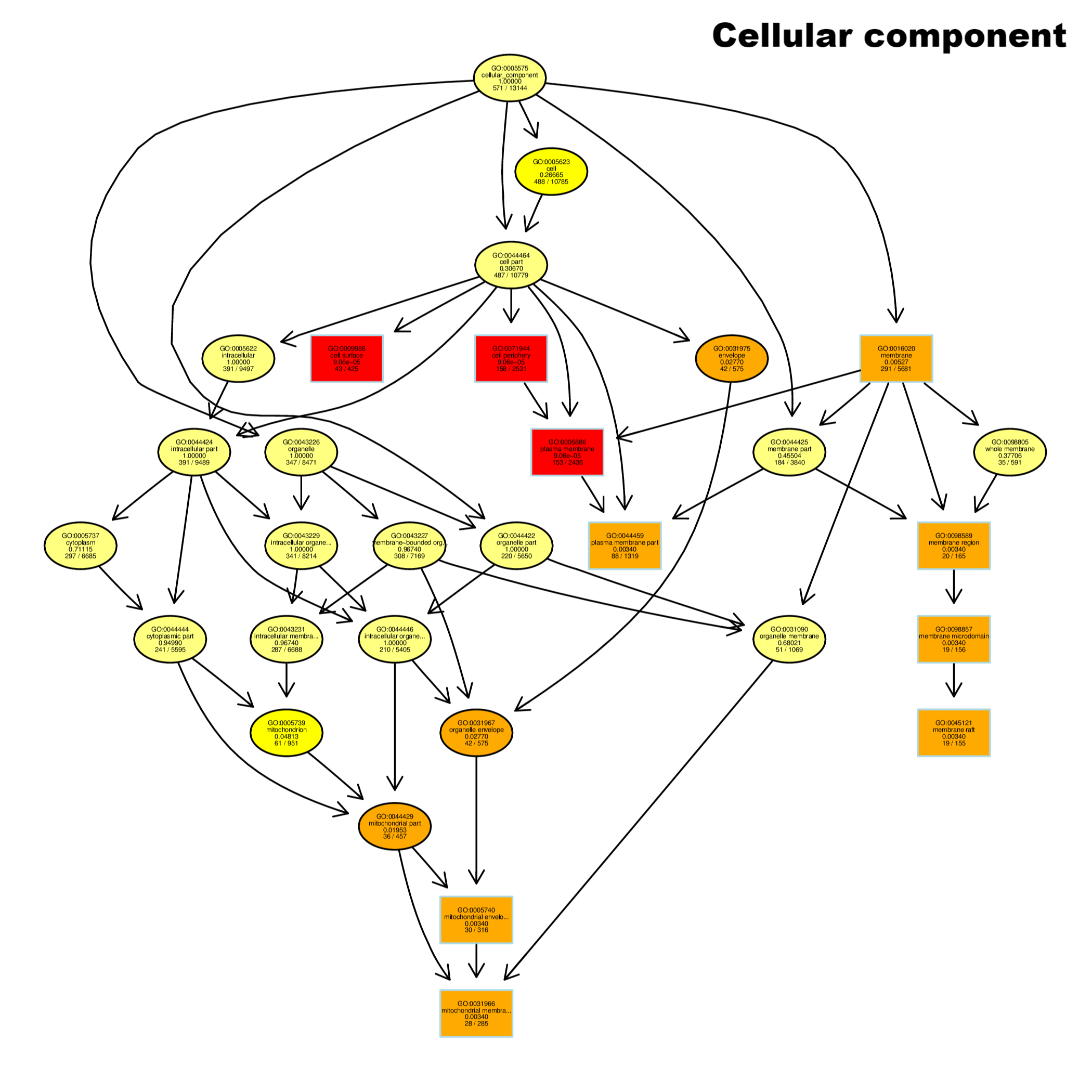


Figure S4. Interaction network corresponding to the enriched GO terms in cellular component.


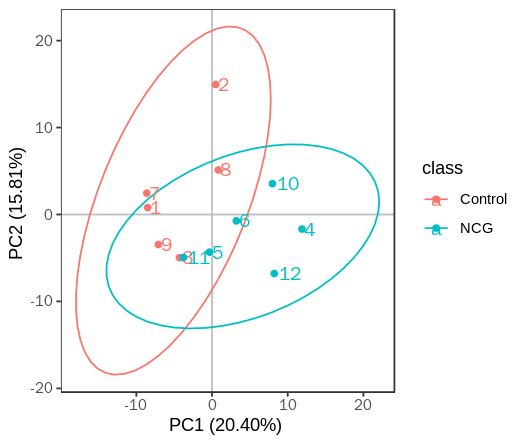

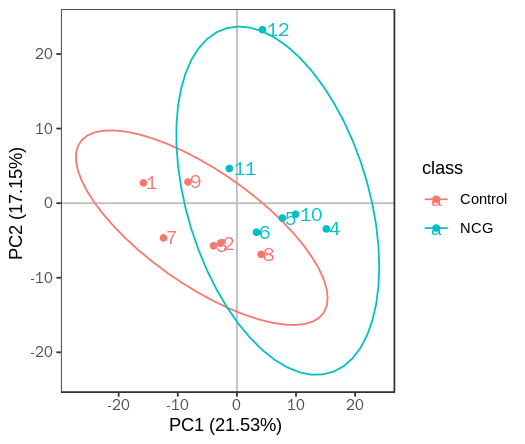


**B**

**A**

Figure S5. The PCA score chart of metabolite profiling data of NCG-vs-Control under the negative ion mode (A) and positive ion mode (B). The difference in metabolism between the two groups is large and the variability between the samples in the group is small.


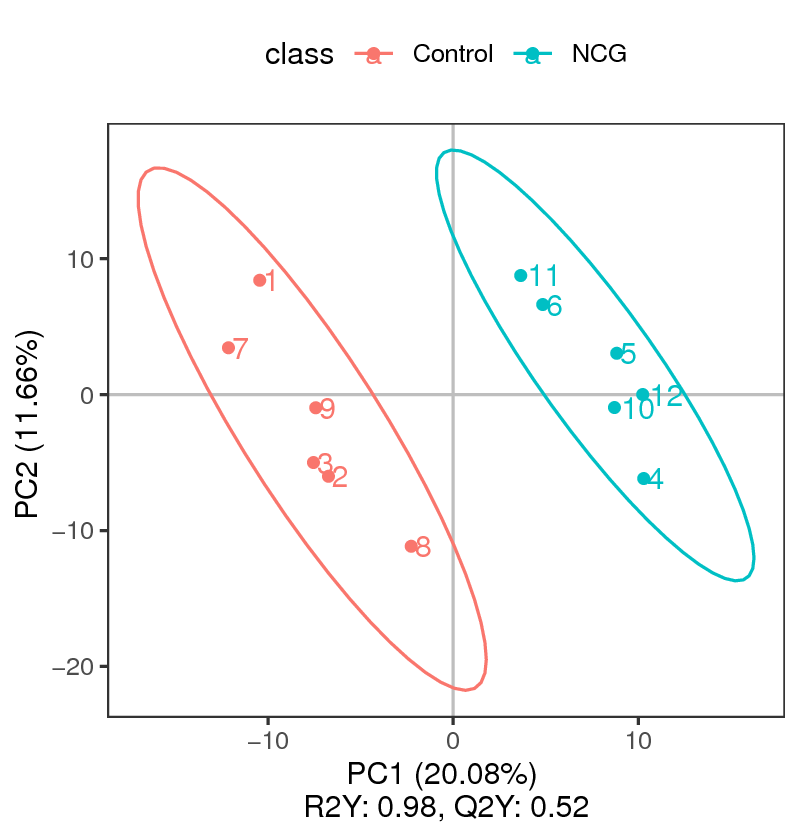

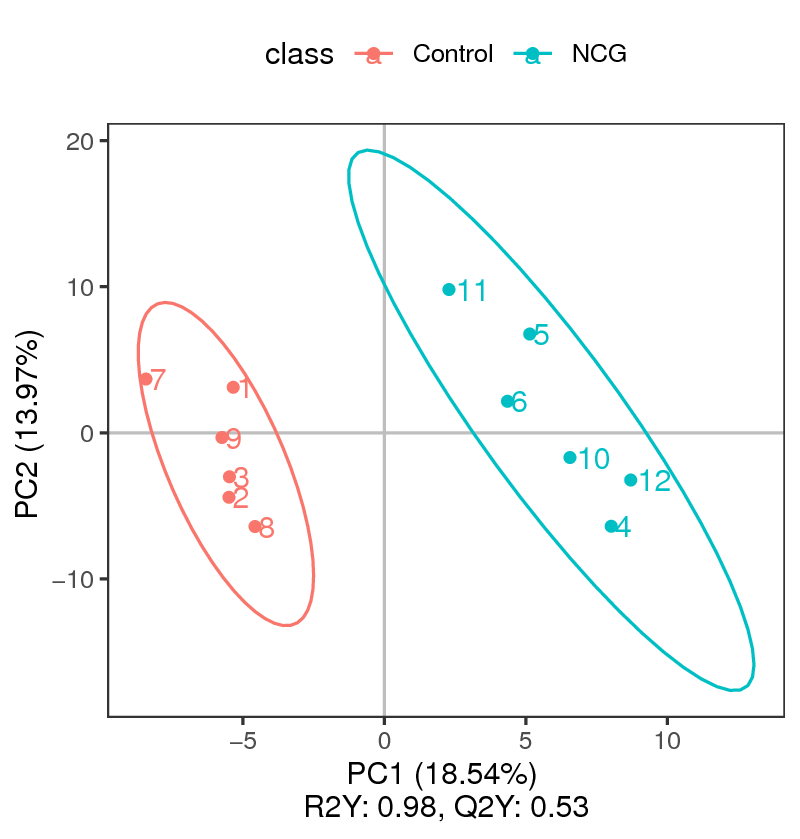


**A**

**B**

Figure S6. The Partial least square-discriminate analysis (PLS-DA) score chart of metabolite profiling data of NCG-vs-Control under the negative ion mode (A) and positive ion mode (B).The difference in metabolism between the two groups is large and the variability between the samples in the group is small.


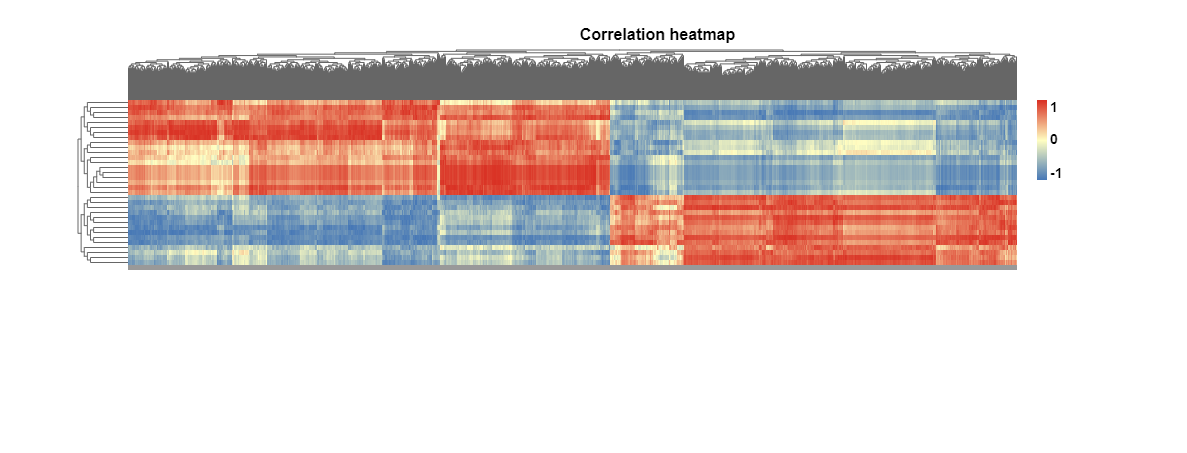


**B**


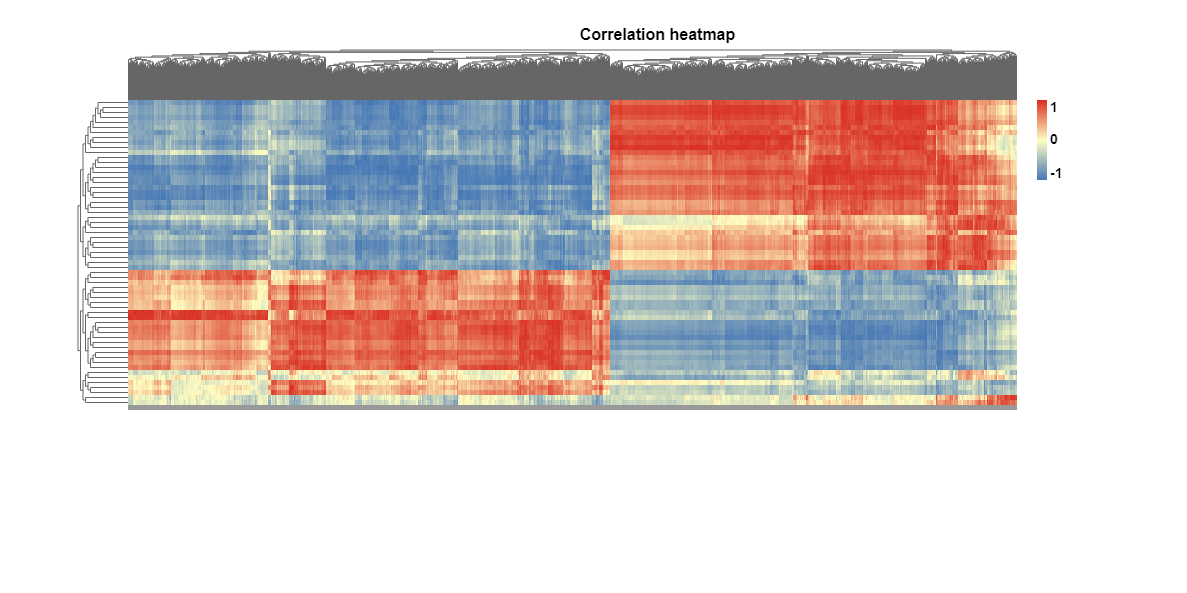


**A**

Figure S7. Integrative analysis using differential metabolites (MDs) and differential expressed genes (DEGs) under the positive analysis ion mode (A) and negative analysis ion mode (B). The horizontal axis shows the clustering of DEGs and the vertical axis indicated the clustering of MDs. The red depth represents the strength of the positive correlation. The blue depth represents the negative correlation.
